# Knee flexion of saxophone players anticipates tonal context of music

**DOI:** 10.1038/s41539-023-00172-z

**Published:** 2023-06-27

**Authors:** Nádia Moura, Marc Vidal, Ana M. Aguilera, João Paulo Vilas-Boas, Sofia Serra, Marc Leman

**Affiliations:** 1 Research Centre for Science and Technology of the Arts, School of Arts, Universidade Católica Portuguesa, Rua de Diogo Botelho 1327, 4169-005 Porto, Portugal; 2 Institute for Psychoacoustics and Electronic Music, Ghent University, Miriam Makebaplein 1, 9000 Ghent, Belgium; 3 Department of Statistics and Institute of Mathematics, Universidad de Granada, Campus de Fuentenueva, 18071 Granada, Spain; 4 Department of Neurology, Max Planck Institute for Human Cognitive and Brain Sciences, Stephanstraße 1a, 04103 Leipzig, Germany; 5 Centre of Research, Education, Innovation and Intervention in Sport (CIFI2D), Porto Biomechanics Laboratory (LABIOMEP-UP), Faculty of Sport, University of Porto, 4099-002 Porto, Portugal

**Keywords:** Human behaviour, Computational neuroscience, Interdisciplinary studies

## Abstract

Music performance requires high levels of motor control. Professional musicians use body movements not only to accomplish and help technical efficiency, but to shape expressive interpretation. Here, we recorded motion and audio data of twenty participants performing four musical fragments varying in the degree of technical difficulty to analyze how knee flexion is employed by expert saxophone players. Using a computational model of the auditory periphery, we extracted emergent acoustical properties of sound to inference critical cognitive patterns of music processing and relate them to motion data. Results showed that knee flexion is causally linked to tone expectations and correlated to rhythmical density, suggesting that this gesture is associated with expressive and facilitative purposes. Furthermore, when instructed to play immobile, participants tended to microflex (>1 Hz) more frequently compared to when playing expressively, possibly indicating a natural urge to move to the music. These results underline the robustness of body movement in musical performance, providing valuable insights for the understanding of communicative processes, and development of motor learning cues.

## Introduction

Multiple mental and sensorimotor processes operate simultaneously underneath the surface of an optimal music performance. The musician’s body plays a critical mediator role between the cognitive processing of musical content and its transformation into the resulting sonic and visual outcomes^1,2^. Research conducted over the last decades has shown that the functions associated with performative body movements go beyond the technical demands of sound production and modification, reaching facilitative, communicative, or expressive purposes^3–9^. The term ancillary gestures was established to define all types of motion, which are not required for playing per se, but potentially support the performance in any other way^9,10^. For example, a saxophone player lifting the instrument’s bell to emphasize expressive intentions, or a singer leaning forward to facilitate reaching a louder sound.

Music, similarly to language, is a complex communication system in which information is conveyed through different sensory channels and not exclusively related to the auditory stimuli^11^. In cognitive linguistics, the power of nonverbal behavior in communicative tasks is not limited to speech comprehension and production^12–16^, but expanded as a tool for enhancing learning processes^14,17–20^. In music research, there is strong evidence that musicians’ body movements affect audience perception^5,21,22^ and reflect features of the musical speech^10,23,24^. However, the knowledge about the facilitative role of gestures in music playing is scarce. Literature suggests movement may aid tempo perception^10,23,25^ and support the execution of dynamics and higher register tones^9,26^. In summary, it seems clear that expert performers associate expression with movement, although they can perform equally good when it is limited^27^. Further research is needed to clarify if ancillary gestures constitute, indeed, an aid to musicians’ technical execution.

Ancillary movement is highly idiosyncratic, as it reflects the individual style of the performer, or speaker^13,28,29^. Nevertheless, gestural trends have been identified among players of the same instrument^10,23,30,31^, and even different instruments^3,29^. Two major factors support this tendency of moving expressively in similar manners: the biomechanical and the musical. Biomechanical factors comprise the technical demands needed to play accurately, in respect to the weight, shape and posture of execution of the instrument. Examples include the technical difficulty of the passages^10,23,24^ and anthropomorphic characteristics of the subjects^26,32^. Musical factors refer to the characteristics of the repertoire being performed, including tempo, dynamics or phrasing structure. Analogously to what occurs with spoken speech^12,13,18,33^, music-related gestures are molded by musical syntax. Musicians’ ancillary movements have been related to phrasing structure^10,34–36^, repeated rhythmic patterns^10,23,37^, and expressive climaxes of the piece^29^. This phenomenon is defined as embodiment - the process of transforming inner subjective experiences, ideas or intentions in a physical manifestation^1,38^.

Finally, the importance of ancillary movement is reinforced by the fact that it emerges, although in smaller scales, when performers are asked to restrict to the minimal movements required for playing^6,10,23^. In this cases, it is argued that movement is so deeply rooted in the performers’ interpretation that it becomes an intrinsic part of the task. On the other hand, several studies support the existence of an innate urge to move to music among listeners when instructed to remain still^39–43^. Significant differences in quantity of motion were found when comparing listeners trying to standstill in silence to when exposed to music^39,40^. In line with these results, involuntary entrainment to groovy music was reported in^41^, and discrete auditory events had a significant effect on involuntary body sway^42^. Thus, movement does not have to be voluntary to enhance cognitive processes related to the perception of complex rhythmic patterns^43^. The fact that movement rises unconsciously only strengthens its role in enhancing cognitive and motor functions implied in quotidian activities.

In this study, we focus on the knee flexion as an ancillary gesture with potential facilitative and expressive functions during saxophone performance. We selected four musical passages of increasing technical difficulty to analyze how rhythmical and tonal components of music were embodied in the performers’ behavior. Each passage was recorded in an immobile (IMO) and an expressive (EXP) condition, as a means to assess to what extent knee movement is an integral part of the performance.

## Results

### Average causal effect between knee flexion and short-term memory tonal expectations

**Fig. 1:**
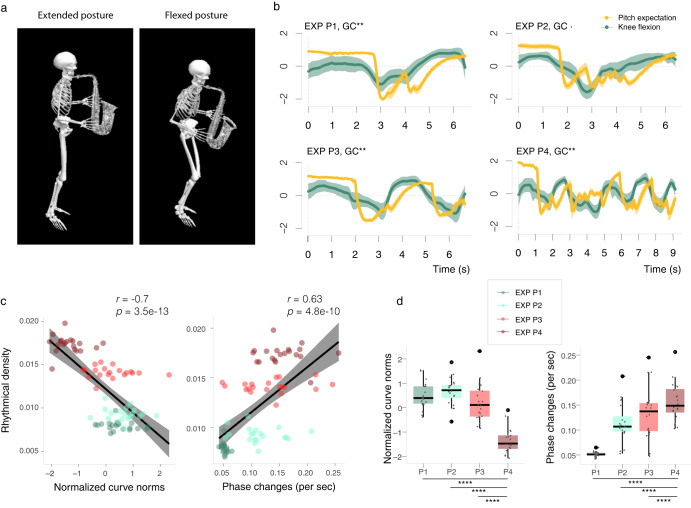
Tonal and rhythmical relations to knee flexion. **a** 3D model of one participant in extended (left) and flexed (right) posture. **b** Comparison of the average Hadamard product curves between right and left knees to pitch expectation profiles (curves were normalized for a better visualization). Granger causality (GC) interactions are noted for a lag equal to 0.15 s onset equivalence; error bands were calculated at a 95% bootstrap confidence interval. **c** The two plots show rhythmical density (vertical axis) compared to: weighted and normalized *L*^2^ norms (area bellow the curve) and phase changes per second of all curves in the expressive condition (EXP). Confidence intervals for the regression line are at 95%. **d** Boxplots showing differences across groups of passages for curve norms and phase changes. Statistical comparisons were made using the Wilcoxon signed-rank test (with Bonferroni-Holm correction for multiple comparisons). The centre line of each boxplot represents the data median and the bounds of the box show the interquartile range. The whiskers represent the bottom 25% and top 25% of the data range–excluding outliers, which are represented by a rounded point. –Significance levels are noted as follows: *p* < 0.1, *p < 0.05, **p < 0.01, ***p < 0.001, ****p < 0.0001, n.s. (not significant).

**Fig. 2:**
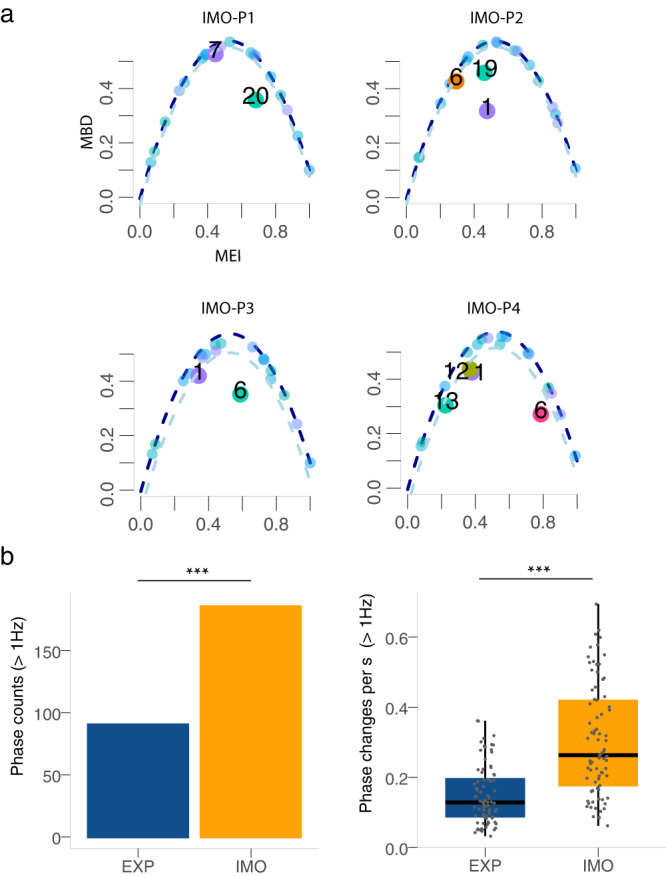
Analyses of participants in immobile condition (IMO) during performance. **a** Functional outlier analysis representation for each passage P1–P4. The vertical axis is the modified band depth (MBD) and the horizontal the modified epigraph index (MEI); larger points and numbers represent outliers related to participants. **b** Boxplots of weighted phase counts and phase changes per second comparing expressive (EXP) and immobile (IMO) conditions. Statistical comparisons were made using the one-sided Wilcoxon signed-rank test (with Bonferroni-Holm correction). The centre line of each boxplot represents the data median and the bounds of the box show the interquartile range. The whiskers represent the bottom 25% and top 25% of the data range. –Statistical significance is measured as detailed in Fig. 1.

We characterized pitch expectation profiles using a computational model of the auditory periphery (see Materials and Methods for technical details). These profiles represent probabilities on incoming pitch under a short-term memory model based on an autocorrelation mechanism that has been described to be coherent with Krumhansl and Kessler experiments on tonal induction^44–46^. Here, we use Granger causality^47,48^ to see if knee flexion has an effect on participant’s internal beliefs of prospective pitch, using a simulated echoic memory buffer generated from their own audio recordings. We performed the analyses on the average of the Hadamard product (element-wise product) between the right and left knee curves and their respective pitch expectation (PE) profiles (both onset aligned, see Materials and Methods) across the four passages (P1–P4). We observed that knee curves could predict pitch expectation as measured by our auditory model (Fig. 1b). The following interactions were found when testing for Granger causality in the EXP condition: P1 [*p* = 0.006, *F* = 2.344], P2 [*p* = 0.063, *F* = 1.702], P3 [*p* = 0.009, *F* = 2.307] and P4 [*p* = 0.002, *F* = 3.30], for a lag calculated as $\lceil (\lambda_{\rm{global}} * \lambda_{\rm{local}}){f}_{\rm{PE}}\rfloor$ ( ≈ [9, …, 12]), where *f*_PE_ is the sampling rate of the pitch expectation profile, *λ*_global_ = 1.5 and *λ*_local_ = 0.1 (in seconds) are model parameters related to the echoic memory (see Materials and Methods). Evidence of this causal relationships was also found in the IMO condition as involuntary flexion was observed: P1 [*p* = 0.024, *F* = 2.025], P2 [*p* = 0.009, *F* = 2.296], P3 [*p* = 0.078, *F* = 1.663] and P4 [*p* < 0.0001, *F* = 4.897]; outliers were removed to perform the tests (Fig. 2a). Further analyses including the left and right knee are provided in the Supplementary Material along with an extensive discussion on the selection of the lag length.

### Saxophone players flex their knees according to rhythmical density

We generated rhythmical density profiles (see Materials and Methods) for each subject and passage (P1–P4) for the EXP condition. The knee curves (Hadamard product) were smoothed using functional multivariate principal component analysis (B-spline dimension = 19, Hubber loss = 48.43, cumulative variance = 99.52 %)^49,50^; this procedure acts as a low-pass filter that takes into account the second order cross-dependencies across conditions. We performed this step to get a neat curvature and to reduce noise that could overlap and bias the estimation of the phase, which was calculated via discrete Hilbert transform. We then estimated the *L*^2^ norms and phase changes per second of the smoothed curves and weighted them by the inverse of the duration of each participant’s performance, to correct for effects produced by the different timings (see Fig. S1a). Pearson correlation coefficient is *r* = − 0.7, *p* < 0.0001 for the *L*^2^ norm scores and *r* = 0.63, *p* < 0.0001 for the phase changes (Fig. 1c) with regard to the rhythmical density scores (*L*^2^ norms of these curves). We also found significant differences in comparing the scores obtained for P4 with regard to the rest of the passages (Fig. 1d).

### Outlier and microflexion analyses suggest natural incline to move in immobile condition

We examined the IMO condition to assess the capacity of participants to restrict their movement. A functional outlier analysis on the knee curves in the IMO condition across passages showed that some curves substantially deviated from a constant angular change (Fig. 2a). To demonstrate the unintentional urge of moving when performing, we conducted further analysis by high-pass filtering the knee curves (three-order Butterworth filter with cutoff at 1 Hz, see Suplementary Material), assuming participants would microflex more often while inhibiting movement than when allowed to move. The analysis of the phase counts revealed that for the IMO condition countings were significantly higher than for the EXP condition (*χ*^2^ = 31.49, *p* < 0.001, using ANOVA via Poisson regression with phase counts weighted according to performance duration). This is also confirmed by the analysis of phase changes per second (*p* < 0.001, Wilcoxon signed-rank test, see Fig. 2b).

## Discussion

Our results explicitly demonstrate a predictive relationship between the averaged knee curves and their respective pitch expectation curves, suggesting movement not only accompanies, but anticipates pitch perception. In predictive coding frameworks^2,51–55^, the principle of active inference implies that the mind is constantly generating models about the environment based on the sensory inputs it receives. By encoding the discrepancy between the expected and the real outcomes of a task, the brain creates a prediction error that potentiates behavioral changes and, therefore, contributes to the refinement of its predictive model. For example, in tapping studies, it is common that participants tap ahead of the metronome^56,57^. Accordingly, the modeled acoustic expectations of our participants enabled them to project their movement in anticipation, finding which is particularly consistent if we take into account that they had previous contact with the piece. We identified several studies reporting correlations between the musical context and musicians’ movements^8,25,35,36^ and, specifically, demonstrating an association between the flexion-extension dynamics and the melodic contour in woodwind instrumentalists^9,29^, which is corroborated by our study. Nevertheless, to our knowledge, the anticipatory movement is a novel result among musicians, which may help understand how gestures can be employed to aid performance. In this sense, we purpose that the motor prediction of the expected auditory outcome may, in fact, facilitate its technical execution. Similarly, research suggests that the coupling of motor and neural processes in multisensory integration contexts may potentiate learning and memory^27,58^. Further research is needed to explore the beneficial effect of embodiment in technical and interpretative aspects of music performing and learning.

Passages with higher rhythmical density (P3–P4) led participants to flex more often, although with smaller motion amplitude, whereas passages with lower rhythmical density (P1–P2) presented larger and less recurrent knee flexions (Fig. 1c, d). Considering that P3–P4 represent higher technical difficulty (due to the fast finger coordination required to play sequences of short-duration notes), the reduction of motion amplitude in these scenarios may be interpreted as a postural strategy with ultimate energy focusing purposes. In technically demanding sections, performers usually reduce their amplitude of motion and limit to focal movements required for accurate execution^10,23,24^. Since knee flexion is not directly related to sound emission in saxophone playing, we purpose that it has an active role in the maintenance of balance and postural control when playing in a standing position.

Previous studies on quiet human stance have demonstrated that the knee joint takes part in the stabilization of the centre of mass^59,60^ and in balance control, in synchronization with the ankle and hip joints^61^. It is now acknowledged that postural control implies multi-joint activation involving the knee, even though the ankle and hip joints are the most activated in this context, and widely studied for decades^62,63^. Postural joint synergy is reinforced by the fact that when the knee joint is constrained, the reliance on ankle strategy increases^64^, and, inversely, when the ankle joint is constrained, adaptation shifts to knee-engaging dynamics^65,66^. Additionally, in the specific case of focal arm movement tasks, which somehow share similarities to the playing task, the activation of leg musculature acts as a compensation of external destabilizing forces^67^.

Microflexions occurred more often during the IMO condition when compared to the EXP, suggesting that saxophone players presented an unintentional inner urge to move during performance, although they were instructed to limit to the minimal movements required for playing. A corpus of research validates music induces involuntary movement, even when subjects try to standstill^39–43^. It is possible that movement is so integrated in the participants’ performative habits that it becomes difficult to erase in a preliminary approach. In order to automatize the playing process, musicians develop motor programs where executive and interpretative functions coexist^6^. After repeated practice of the same piece, these motor programs become ingrained and hard to remove^6,10^. Small movements have emerged before in performances with movement restriction^6,10,23,68^. Nonetheless, in order to further strengthen this finding, it would be pertinent to compare the immobile condition with several kinds of resting state behavior (e.g., holding or not the instrument, etc.) or behavioral data assessment.

Preserving the natural expressive behavior of the participants was a major consideration in our design. For that, we used musical excerpts from standard saxophone repertoire, assuring high degrees of familiarity with the interpreted music. The passages were selected considering a criteria of redundancy, which allowed for fair in-between comparisons. In this sense, the musical contexts we studied were characterized by starting with descendant pitch lines and presenting low rhythmical density associated with higher pitches. Follow-up studies including other variations in pitch, rhythm, but also structure or articulation, are necessary to clarify the role of knee flexion in diverse musical contexts. Similarly, other musical styles and genres should be considered.

In conclusion, our study provides novel evidence about the predictive role of the sensorimotor system in music performance. We purpose that anticipatory knee movement facilitates the cognitive processing involved in motor adaptation, and consequently supports technical execution. This, in addition to the correlations found between rhythmical density profiles of the music and phase and amplitude of knee movement, and the underlying urge to move in immobile conditions, reinforces the central role of the body in musical expression. We suggest these results constitute a valuable contribution for other fields involving communication, such as the performing arts, linguistics, or human-computer interaction. Furthermore, this study provides insights for the development of motor learning programs focused on proprioceptive and kinaesthetic awareness.

## Methods

### Participants

The recordings used for this study were retrieved from the database reported in ref. ^69^, including a sample of 20 participants (9 female, 11 male; mean age: 26.3 years ± 5.4 years; mean duration of saxophone practice: 16.9 years ± 5.6 years). This study was approved by the Ethics Committee for Health of Universidade Católica Portuguesa (protocol number 137/2021), and the procedures were carried out according to the Declaration of Helsinki. Prior to the recording session, all participants were informed about the data collection and protection protocols, signed a written informed consent form and were sent the repertoire for preparation. Also, at this point, we confirmed that they had already studied the piece before.

### Musical repertoire

The musical passages used for this study were extracted from the piece Concertino da Camera for solo saxophone and orchestra, by Ibert^70^. They were selected as representative of the two musical contexts planned for our study: melodic lines comprising an interval jump, and technical passages with rhythmical density. Two passages per context were chosen for redundancy purposes, resulting in a total of four passages for analysis. Music scores and analyses are presented in the Supplementary Material.

### Data collection

Motion data was recorded with an optical 3D motion capture system with 9 infrared cameras, at a sampling frequency of 240 Hz. The marker setup adopted included 58 retroreflective markers placed in the body of the subject and 9 markers placed in the saxophone (for more details, see ref. ^69^). Motion data was then processed using Qualisys Track Manager 2021 (QTM). Knee angular values were extracted from QTM at a sampling rate of 240 Hz, by calculating the angle between the lateral markers of the right and left ankle, knee and hip. Audio data was recorded with a Zoom H4N recorder, at a frequency rate of 44.1 Hz. Additionally, to align all data channels, we recorded video using a Canon EOS 100D with a 18-55mm lens, introducing an audiovisual signal with a clapperboard in the beginning and end of each take (2 markers attached). We then synchronized timestamps for the multiple devices retrospectively in respect to this signal.

In the recordings, participants were asked to play in two conditions: immobile (restricting their movement to the minimum required for effective playing) and expressive (moving as idealized in a concert situation). Conditions and passages were randomized. Players had the freedom to perform in the tempo they found appropriate to their interpretation.

### Curve registration using onset positions in the audio signal

**Fig. 3:**
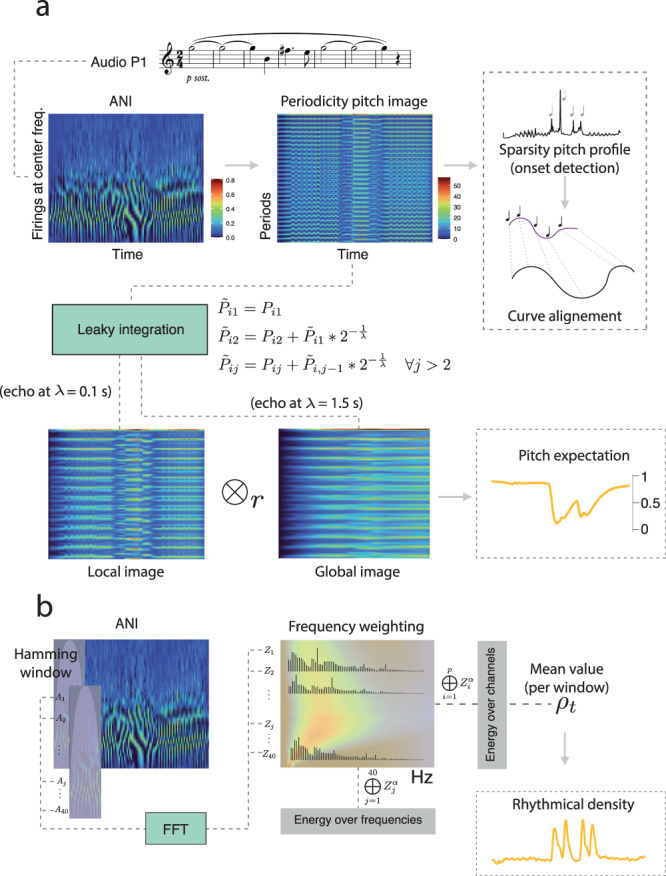
Schematic of the auditory modeling framework. **a** The auditory nerve image (ANI) is estimated from a mono audio signal and then converted into periodicity pitch image from which we extract different pitch profiles for further analysis. Leaky integration is performed for echoes of 0.1, 1.5 s on the periodicity pitch image to obtain two images of different echo whose Pearsons' correlation index between running columns gives the pitch expectation profile. **b** Calculation of the rhythmical density from ANI. The operator ⊕ is used to denote the column/row sums of the matrix *Z*^*α*^.

Here, we describe the procedure to align the knee curves to a set of audio features by transforming their abscissa variables. Registration was conducted by retrieving strategic note onsets (see Supplementary Fig. 2 in the Supplementary Material) from the audio using computational modeling of the human auditory periphery (Fig. 3a). The challenge here is the homogeneity of tone changes. The merging of fundamental and harmonic frequencies in the spectral envelope, along with small oscillations in the air column that are characteristic of the saxophone acoustic signal^71^ made it difficult to detect note onsets with some existent methods.

To simulate the auditory brainstem response to sound, the acoustic signal is firstly converted into a multivariate pattern of neural impulses^72,73^. The algorithm generates, from a mono audio signal *s*_*t*_ (*t* = 1, …, *N*), a matrix (or auditory nerve image, ANI) $A\in {{\mathfrak{R}}}^{q\times n}$ with *q* = 40 channels representing the instantaneous amplitude across frequency sub-bands half a critical band apart from each other, with center frequencies on the range 140 Hz to 8877 Hz. Thus, *A* stands for a time-frequency representation with frequencies spanning the audible range, capturing the information in *s*_*t*_ as an auditory precept that simulates the peripheral auditory system function. The ANI sampling rate was determined as *f*_ANI_ = 11025/4, so that $n=(N* {f}_{{{{\rm{ANI}}}}}){f}_{s}^{-1}$, where *f*_*s*_ denotes the sampling rate of *s*_*t*_.

A second order Butterwoth band-pass filter (80–1250 Hz) is applied to each row of the ANI matrix ${A}_{i}\to {\tilde{A}}_{i}\,(i=1,\ldots ,q)$ to retrieve pitch periodicity patterns. Then, a frame-based convolution analysis and a coincidence mechanism are conducted by the summation of the convolution results over all channels. For a fixed frame width *δ* (usually of 60 ms^44^) and frame lag *w* (10 ms), each column of the periodicity pitch image is obtained from a filtered ANI image $\tilde{A}$ as

1 $$\begin{array}{lll}{P}_{1}\,=\,\mathop{\sum }\limits_{i=1}^{q}{\tilde{A}}_{i,1:\delta } \circledast {\tilde{A}}_{i,(\delta +1):2\delta }\\ {P}_{2}\,=\,\mathop{\sum }\limits_{i=1}^{q}{\tilde{A}}_{i,(w+1):(w+\delta )} \circledast {\tilde{A}}_{i,(w+\delta +1):(w+2\delta )}\\ \qquad\vdots \,\\ {P}_{j}\,=\,\mathop{\sum }\limits_{i=1}^{q}{\tilde{A}}_{i,((j-1)w+1):((j-1)w+\delta )} \circledast {\tilde{A}}_{i,((j-1)w+\delta +1):((j-1)w+2\delta )},\end{array}$$

for all *j* ≤ *w*^−1^(*n* − 2*δ*) + 1, (*j* = 1, …, ⌈*w*^−1^(*n* − 2*δ*) + 1⌉). Note that the periodicity pitch image represents estimated periodicities in the firing patterns of the ANI. Then, to estimate pitch onsets, a sparsity profile is defined as

2 $${\kappa }_{j}={{{\rm{kurt}}}}({P}_{j})=\frac{\mathop{\sum }\nolimits_{i = 1}^{\delta }{({P}_{ij}-{\overline{P}}_{j})}^{4}}{{\left(\mathop{\sum }\nolimits_{i = 1}^{\delta }{({P}_{ij}-{\overline{P}}_{j})}^{2}\right)}^{2}}.$$

The resulting time series peaks at image times of maximum kurtosis, meaning that it captures transitions from tone to tone based on the assumption that periodicities decrease at high velocity in such locations. For this particular case, we constructed ANI using first differences in *s*_*t*_, as derivatives are natural tools for pre-whitening, which is commonly used to enhance the estimation of fourth-order moments^74^. Then, to detect the note onsets, we select a number of expected outcomes and find iteratively maximas in *κ*_*j*_ by defining the threshold ${\overline{\kappa }}_{j}* (1.5-0.001)* {{{\rm{SD}}}}({\kappa }_{j})$ with a decreasing factor of 0.001 until the expected number of peaks is achieved.

After the pitch onsets were detected, auditory checking and manual correction was performed in SonicVizualizer^75^. Performance metrics were derived: about 92.40%, 71.66%, 76.80%, and 76.875% of correct onsets were achieved for P1–P4, respectively.

Curve registration was conducted by aligning the abcissa variables of P1–P4 group of curves to their respective vector of mean onsets by resampling each snippet to (240*⌈onset⌋)*ℓ*^−1^ where *ℓ* is the length for each snippet and ⌈onset⌋ is a vector of rounded mean onsets of size the number of expected onsets per passage. All curves were finally sampled to a fine grid of equal time points.

### Pitch expectation index

This index measures the pitch commonality using two running leaky integrated periodicity pitch images, each one having a possible different echo (Fig. 3a). Local images (short echo) are compared with the global images (long echo) at run time. This comparison generates tone expectations based on constant updates according to the spectral properties related to changes in pitch. The formal expression for this index is

3 $${\tilde{P}}_{{\lambda }_{{{{\rm{local}}}}}}{\otimes }_{r}{\tilde{P}}_{{\lambda }_{{{{\rm{global}}}}}}\to [-1,\ldots ,1],$$

where $\tilde{P}$ is a leaky integrated pitch image, *λ* stand for the respective echoes in seconds (*λ*_local_ = 0.1 for local, *λ*_global_ = 1.5 for global) and ⊗ _*r*_ is an operator that calculates the Pearsons’ correlation coefficient between running columns of both matrices. Echoes durations’ are determined according to the short-time memory model formulated in ref. ^44^. Leaky integration is exemplified for a null factor of enlargement as detailed in Fig. 3a.

In summary, given a sequence of pitches, the pitch expectation index shows a curve of correlations going up and down, depending on how well the subsequent individual pitches and the previous pitches (in a given time window) match.

### Rhythmical density

Here we define rhythmical density (*ρ*) by the energy of relevant beating frequencies over each ANI row (channels). As shown in Fig. 3b, a set of (*k*) ANI overlapping frames mapped by a Hamming window are transformed to the frequency domain per channel using fast discrete Fourier transform (FFT). We obtain a matrix of size *q* × *p*, where *p* is the length of the spectrum, that is weighted more prominently at channel center frequencies bellow 800 Hz and attenuated for high spectral frequencies as well, to reproduce psychoacoustic sensation of roughness or dissonance, a critical dimension of timbre^76^. Without loss of generality, the weighed matrix $Z\in {{\mathfrak{R}}}^{q\times p}$ is usually exponentiated at *α* = 1.6 by the power law. The sums of the weighted bins across each channel produces a vector 1 × *q* whose mean value is the rhythmical density *ρ*_*t*_ for each ANI frame (*t* = 1, …, *k*). Rhythm is therefore represented continuously according to the roughness properties of sound which makes this index particularly sensitive to pitch changes when there is homogeneity in timbre. Note that this index increases and keeps sustained according to pitch appearance and decays regardless of their duration and volume.

### Reporting summary

Further information on research design is available in the Nature Research Reporting Summary linked to this article.

## Supplementary information

Supplementary Material

Reporting Summary

Supplementary Movie S1

## Data Availability

Restrictions apply to the availability of these data, which were used under licence for the current study and so are not publicly available. However, anonymized motion and audio data that support the findings of this study are available from the corresponding author N.M. upon reasonable request for research purposes.

## Code availability

The routines for the computational modeling of the auditory periphery are freely available as an R-package at a Github repository^74^. ANIs are computed with the function CalcANI, periodicity pitch images with PeriodicityPitch, pitch expectation profiles with ContextualityIndex (Comparison) and rhythmical density with RoughnessFFT.
