# Supplementary material for: Knee flexion of saxophone players anticipates tonal context of music
Source: NPJ Sci Learn. 2023 Jun 27;8:22. doi: 10.1038/s41539-023-00172-z (PMC10300100; doi:10.1038/s41539-023-00172-z)
Supplement: Supplementary file 1 — Supplementary Material [file 41539_2023_172_MOESM1_ESM.pdf]

Supplementary Information for "Knee flexion of saxophone players anticipates tonal context of music"

Nádia Moura*[1†], Marc Vidal*[2,3,4†], Ana M. Aguilera[3], João Paulo Vilas-Boas[5], Sofia Serra[1], and Marc Leman*[2]

[1]Research Centre for Science and Technology of the Arts, School of Arts, Universidade Católica Portuguesa, Rua de Diogo Botelho 1327, 4169-005 Porto, Portugal
[2]Institute for Psychoacoustics and Electronic Music, Ghent University, Miriam Makebaplein 1, 9000, Ghent, Belgium
[3]Department of Statistics and Institute of Mathematics, Universidad de Granada, Campus de Fuentenueva, 18071, Granada, Spain
[4]Department of Neurology, Max Planck Institute for Human Cognitive and Brain Sciences, Stephanstraße 1a, 04103, Leipzig, Germany
[5]Centre of Research, Education, Innovation and Intervention in Sport (CIFI2D), Porto Biomechanics Laboratory (LABIOMEP-UP), Faculty of Sport, University of Porto, 4099-002 Porto, Portugal
[*]corresponding author(s): Nádia Moura (nmoura@ucp.pt)
[†]first co-authors

ABSTRACT

Supplementary material include: Supplementary Additional Information; Supplementary Figures S1, S2; Supplementary Table S1; Supplementary Movie S1.

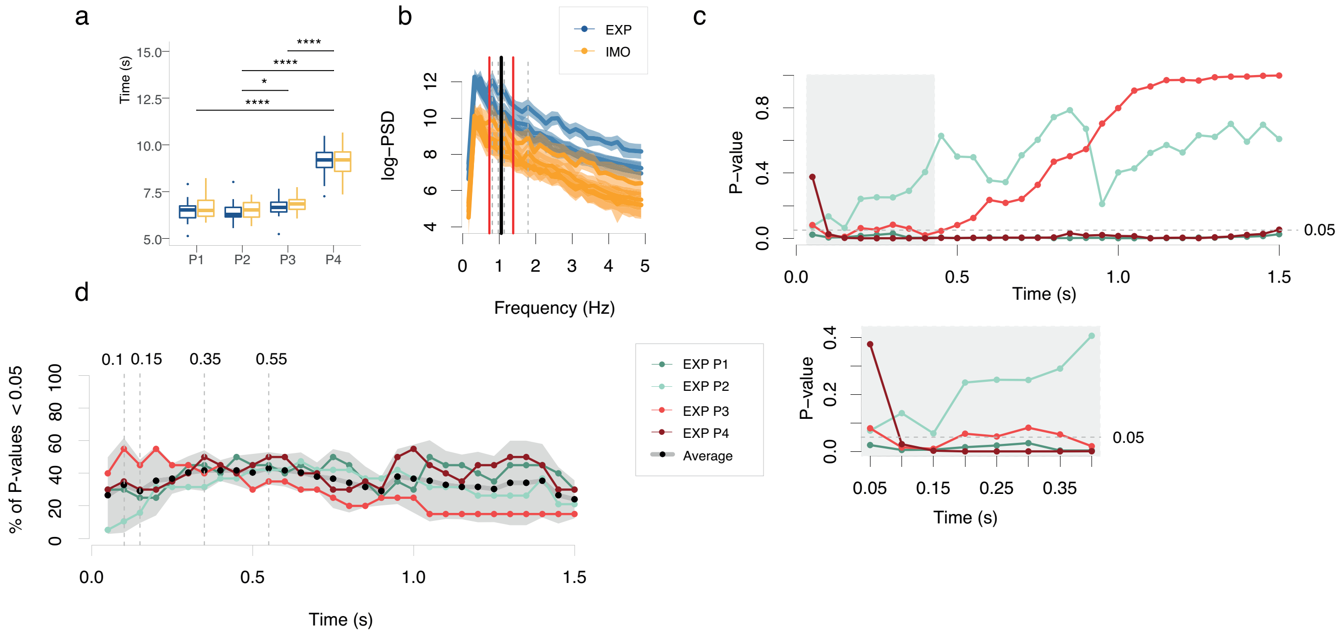

Supplementary Figure 1. Miscellania. a Boxplots of the time durations of the passages showing statistical differences between them using the Wilcoxon signed-rank test (with Bonferroni-Holm correction for multiple comparisons). The centre line of each boxplot represents the data median and the bounds of the box show the interquartile range. The whiskers represent the bottom 25% and top 25% of the data range—excluding outliers which are represented by a rounded point. **b** Spectral analysis (in logarithmic scale) comparing the different passages across conditions to determine the initial higher frequency threshold for knee motion data. The (vertical) dashed grey lines represent the second peak in frequency of the curves whereas the black line is the mean of all of them (the red lines are the standard deviation around this mean, 1.059 ± 0.326). **c** Granger causality (GC) results between averaged pitch expectation and knee curves on a time grid $\{0.05, 0.1, \ldots, 1.5\}$. **d** GC analysis across participants (average values) and passages measuring in % the global significance level ($p < 0.05$) of causality between marginal curves. Confidence band relate to percentage values of the four passages. - All error bands were calculated at a 95% bootstrap confidence interval.

A cautionary note on measuring Granger causality between pitch expectation profiles and knee curves

As it has been previously reported, a suitable selection of the lag-length is crucial to avoid the so-called spurious causalities[1,2]. We used an approach that combines both local (0.1 s) and global (1.5 s) echoes to determine the optimal time-lag for Grangers' causality (GC) analysis. The rationale is straightforward: the ratio that defines the global echo in relation to the local one provides the reference lag, that is, $\lambda_{\text{global}} = \text{lag}/\lambda_{\text{local}}$. The optimality of this choice (EXP condition) is shown in Fig. S1*c* (GC over averaged curves), where it can be observed that the joint p-value level across conditions reaches maximal significance at time-lag 0.15 s. We could partially confirm this using the Akaike information criterion (AIC) and the Bayesian information criterion (BIC), as these criteria tend, respectively, to overestimate and underestimate the lag-length[2]. Bellow 0.05 s lag, we found GC analyses on mean curves were often biased due to substantial autocorrelation in the residuals (Portmanteau test) while Granger's principle that "the effect does not precede the cause in time" did not hold. Therefore, in order to improve AIC/BIC estimation we removed the 5 first onset-lags, and perform again the analyses: the average results for the four curves evaluated for $\{6, \ldots, 100\}$ suggested an onset-lag of 10.5 (average of both criteria) which corresponds to a time-lag of 0.150 (calculated using the mean of all timings). This last step was performed taking logarithms and first differences in all mean curves (otherwise the common procedure gives a time-lag equal to 0.157).

We further calculated the mean percentages of p-values < 0.05 across all subjects, which are also shown in Fig. S1*d* pooled by condition. Comparing with the results in Fig. S1*c* (see also Tab. S1 for a detailed account on the p-values), it can be argued that motor adaptation to pitch expectations might occur from $\sim$0.05 to 0.55 s, where maximal percentage across participants is reached (43.03%, Fig. S1*d*). In such ecological conditions, one cannot expect knee flexion to be homogeneous across participants, neither linearly related to pitch expectation (other musical/non-musical actions play a role during the performance). This might explain the percentages in Fig. S1*d* and, for instance, the high sensitivity to lag variations (<0.5 s) in EXP P3 (Fig. S1*c*). Therefore we assumed, similarly as in event-related potential analysis, that averaging suppresses non-phase-locked activity thus revealing the true shape of the process. In other words, under mild tonal conditions, the mean (or first moment)

Time-lag (s)	Condition	p-value		F-statistic
0.1	EXP P1	0.004	**	2.792
0.1	EXP P2	0.134	n.s.	1.56
0.1	EXP P3	0.014	*	2.54
0.1	EXP P4	0.025	*	2.584
0.15	EXP P1	0.006	**	2.344
0.15	EXP P2	0.063	·	1.702
0.15	EXP P3	0.009	**	2.307
0.15	EXP P4	0.001	**	3.155
0.35	EXP P1	0.003	**	1.965
0.35	EXP P2	0.291	n.s.	1.136
0.35	EXP P3	0.059	·	1.489
0.35	EXP P4	0.000	***	2.615
0.55	EXP P1	0.002	**	1.812
0.55	EXP P2	0.497	n.s.	0.987
0.55	EXP P3	0.124	n.s.	1.278
0.55	EXP P4	0.002	**	1.92

Supplementary Table 1. Analyses of time lags for GC tests between averaged knee and pitch expectation curves (Fig. S1*c*).

provides the sufficient information to claim that knee flexion is valuable to enhance expectation on prospective pitch. This interpretation paves the way to explore non-linear relations between motion data and other musical parameters possibly using nonlinear kernel/functional methods.

Trend stationary was warranted in all mean curves ($p < 0.01$, Kwiatkowski-Phillips-Schmidt-Shin test) which allowed to test GC under weak stationarity. Only for P4 we found signs of non-stationarity ($p = 0.064$). Curves were de-trended where necessary in all of our analyses. GC tests were conducted using the `grangertest` function of the `R`-package `lmtest`.

GC analyses per knee

We additionally report the GC results for both knees using a time-lag of 0.15. We found the following interactions for the right knee in the EXP condition: P1 [$p = 0.2908, F = 1.185$], P2 [$p = 0.0747, F = 1.702$], P3 [$p = 0.009, F = 2.310$] and P4 [$p = 0.003, F = 2.934$]; and for the left knee: P1 [$p = 0.000, F = 4.098$], P2 [$p = 0.129, F = 1.475$], P3 [$p = 0.016, F = 2.139$] and P4 [$p = 0.002, F = 2.992$].

Selection of the initial higher frequency threshold for knee flexion motion data

We normalized the sampling rate of all knee curves. Further, we performed the fast Fourier transform and derived the log spectrum (log-PSD) up to 5 Hz (Fig. 1*b*). For each passage across conditions we calculated the averaged log-PSD's (6 curves in total) and then estimated the second peak of these curves, as we observed the first one (mean=0.387 ± 0.084 Hz) was clearly related to wide movements of the knee. The mean frequency for this second peak was 1.059 ± 0.326. This result is not surprising, since the starting point defining the high-frequency activity of various slow phenomena, such as seismic waves[3] or cognitive-related pupil activity[4], is around 1 Hz. Accordingly, we used 1 Hz in our analyses.

Supplementary Movie S1

Multimodal visualization of the data channels used in this study, presenting one example per passage and condition, including: video of the 3D model of one participant, corresponding musical score, knee angle, tonal expectation and rhythmical density curves.

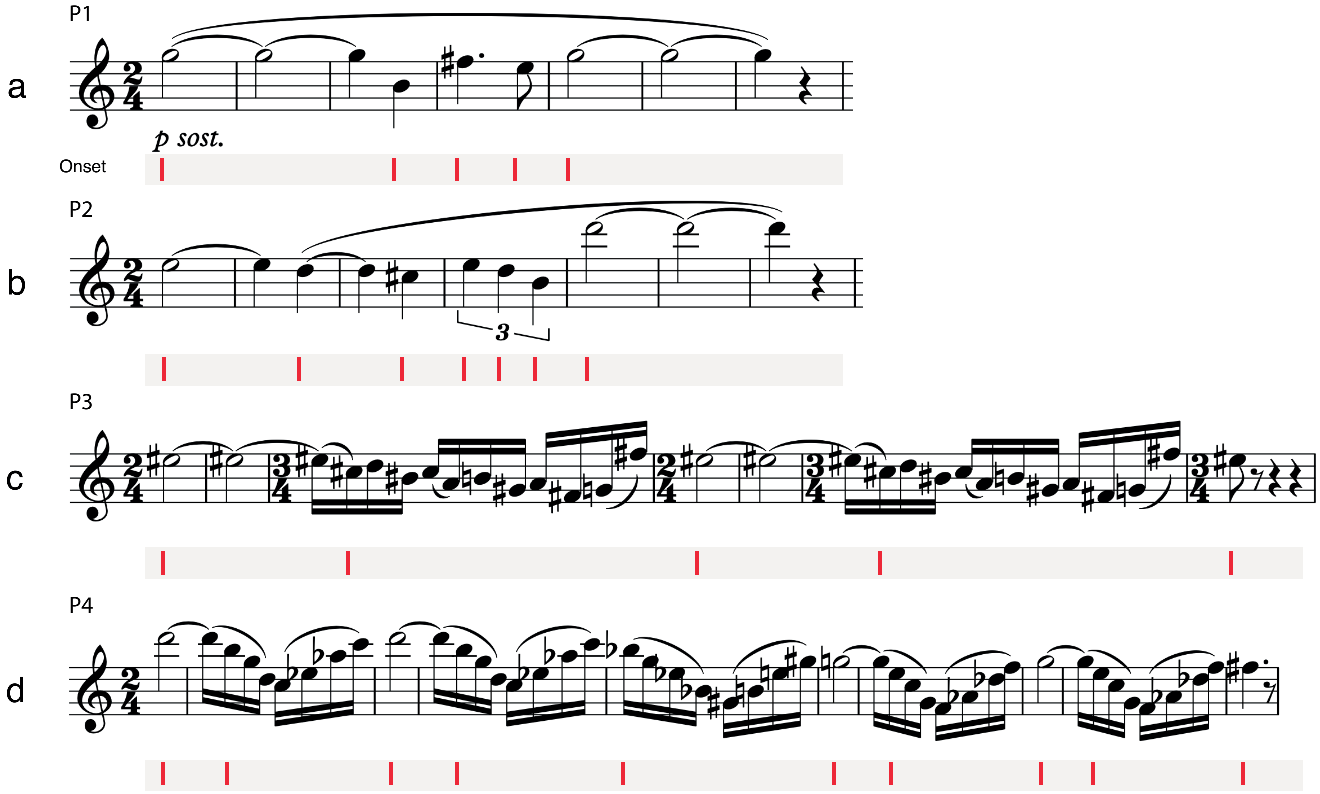

Supplementary Figure 2. Musical passages included in this study, extracted from Concertino da Camera by Ibert[5]. The red lines represent the note onsets detected per passage. **a** Passage 1 (bars 65-71). Melodic line, legato, no rhythmical density, pitch follows a descending-ascending gesture. **b** Passage 2 (bars 77-83). Melodic line, legato, no rhythmical density, pitch follows a descending-ascending gesture. **c** Passage 3 (bars 95-101). Passage containing two fragments of long duration notes interpolated with rhythmical density. Bars 3 and 6 are technically demanding. **d** Passage 4 (bars 103-112). Passage containing four fragments of long duration notes interpolated with rhythmical density. Bars 2, 4, 5, 7 and 9 are technically demanding.

References

1. Eichler, M. Causal inference with multiple time series: principles and problems. *Philos. Trans. Royal Soc. Ser. A* **371** (2013).

2. Bruns, S. B. & Stern, D. I. Lag length selection and p-hacking in granger causality testing: prevalence and performance of meta-regression models. *Empir. Econ.* **56**, 797–830 (2018).

3. D'Amico, S., Akinci, A. & Pischiutta, M. High-frequency ground-motion parameters from weak-motion data in the Sicily Channel and surrounding regions. *Geophys. J. Int.* **214**, 148–163 (2018).

4. Vidal, M. *et al.* Cholinergic-related pupil activity reflects level of emotionality during motor performance. *Eur. J. Neurosci.* https://doi.org/10.1111/ejn.15998.

5. Ibert, J. *Concertino da camera pour Saxophone et orchestre* (Alphonse Leduc Éditions Musicales, 1935).
