# Supplementary material for: Knee flexion of saxophone players anticipates tonal context of music
Source: NPJ Sci Learn. 2023 Jun 27;8:22. doi: 10.1038/s41539-023-00172-z (PMC10300100; doi:10.1038/s41539-023-00172-z)
Supplement: Supplementary file 2 — Reporting Summary [file 41539_2023_172_MOESM2_ESM.pdf]

nature portfolio

Corresponding author(s): Nádia Moura

Last updated by author(s): Nádia Moura

Reporting Summary

Nature Portfolio wishes to improve the reproducibility of the work that we publish. This form provides structure for consistency and transparency in reporting. For further information on Nature Portfolio policies, see our Editorial Policies and the Editorial Policy Checklist.

Statistics

For all statistical analyses, confirm that the following items are present in the figure legend, table legend, main text, or Methods section.

n/a	Confirmed	
☐	☒	The exact sample size (*n*) for each experimental group/condition, given as a discrete number and unit of measurement
☐	☒	A statement on whether measurements were taken from distinct samples or whether the same sample was measured repeatedly
☐	☒	The statistical test(s) used AND whether they are one- or two-sided<br>*Only common tests should be described solely by name; describe more complex techniques in the Methods section.*
☐	☒	A description of all covariates tested
☐	☒	A description of any assumptions or corrections, such as tests of normality and adjustment for multiple comparisons
☐	☒	A full description of the statistical parameters including central tendency (e.g. means) or other basic estimates (e.g. regression coefficient) AND variation (e.g. standard deviation) or associated estimates of uncertainty (e.g. confidence intervals)
☐	☒	For null hypothesis testing, the test statistic (e.g. *F*, *t*, *r*) with confidence intervals, effect sizes, degrees of freedom and *P* value noted<br>*Give P values as exact values whenever suitable.*
☒	☐	For Bayesian analysis, information on the choice of priors and Markov chain Monte Carlo settings
☐	☒	For hierarchical and complex designs, identification of the appropriate level for tests and full reporting of outcomes
☐	☒	Estimates of effect sizes (e.g. Cohen's *d*, Pearson's *r*), indicating how they were calculated

Our web collection on statistics for biologists contains articles on many of the points above.

Software and code

Policy information about availability of computer code

Data collection	Code was not used for data collection.
Data analysis	All analyses were conducted using custom R scripts available from the corresponding author (MV) under reasonable request. The routines for the computational modeling of the auditory periphery are freely available as an R package at a Github repository. ANIs are computed with the function CalcANI, periodicity pitch images with PeriodicityPitch, pitch expectation profiles with ContextualityIndex (Comparison) and rhythmical density with RoughnessFFT.

For manuscripts utilizing custom algorithms or software that are central to the research but not yet described in published literature, software must be made available to editors and reviewers. We strongly encourage code deposition in a community repository (e.g. GitHub). See the Nature Portfolio guidelines for submitting code & software for further information.

Data

Policy information about availability of data

All manuscripts must include a data availability statement. This statement should provide the following information, where applicable:

- Accession codes, unique identifiers, or web links for publicly available datasets
- A description of any restrictions on data availability
- For clinical datasets or third party data, please ensure that the statement adheres to our policy

Motion and audio data that support the findings of this study are available from the corresponding author (NM) upon reasonable request.

Research involving human participants, their data, or biological material

Policy information about studies with human participants or human data. See also policy information about sex, gender (identity/presentation), and sexual orientation and race, ethnicity and racism.

Reporting on sex and gender	Although we collected data on the biological sex of the participants based on their self-reports (presented in the Methods), this was not a variable of interest for the present study, so no further analyses were conducted.
Reporting on race, ethnicity, or other socially relevant groupings	We did not collect data about socially relevant groupings, as this was not a variable of consideration in our study.
Population characteristics	See above.
Recruitment	Participants were recruited by email invitation, considering the criteria of a) having completed or currently pursuing undergraduate or graduate studies in saxophone performance, b) having a minimum of 10 years of formal saxophone practice, c) having previous knowledge of the piece to be recorded.
Ethics oversight	This study was approved by the Ethics Committee for Health of Universidade Católica Portuguesa (protocol number 137/2021).

Note that full information on the approval of the study protocol must also be provided in the manuscript.

Field-specific reporting

Please select the one below that is the best fit for your research. If you are not sure, read the appropriate sections before making your selection.

☐ Life sciences ☒ Behavioural & social sciences ☐ Ecological, evolutionary & environmental sciences

For a reference copy of the document with all sections, see nature.com/documents/nr-reporting-summary-flat.pdf

Life sciences study design

All studies must disclose on these points even when the disclosure is negative.

Sample size	*Describe how sample size was determined, detailing any statistical methods used to predetermine sample size OR if no sample-size calculation was performed, describe how sample sizes were chosen and provide a rationale for why these sample sizes are sufficient.*
Data exclusions	*Describe any data exclusions. If no data were excluded from the analyses, state so OR if data were excluded, describe the exclusions and the rationale behind them, indicating whether exclusion criteria were pre-established.*
Replication	*Describe the measures taken to verify the reproducibility of the experimental findings. If all attempts at replication were successful, confirm this OR if there are any findings that were not replicated or cannot be reproduced, note this and describe why.*
Randomization	*Describe how samples/organisms/participants were allocated into experimental groups. If allocation was not random, describe how covariates were controlled OR if this is not relevant to your study, explain why.*
Blinding	*Describe whether the investigators were blinded to group allocation during data collection and/or analysis. If blinding was not possible, describe why OR explain why blinding was not relevant to your study.*

Behavioural & social sciences study design

All studies must disclose on these points even when the disclosure is negative.

Study description	This study investigates the relationships between musicians' movement (specifically, knee flexion) and tonal and rhythmical components of the music, with the ultimate goal of contributing to the understanding of the sensorimotor processes underlying music performing. All data collected are quantitative.
Research sample	Twenty expert saxophone players (sex: 9 females; mean age: 26.3 ± 5.4 years old). Musical expertise was measured considering the years of formal saxophone practice (mean: 16.9 ± 5.9 years).
Sampling strategy	We used convenience sampling, as participants were selected from the music professional network of NM and invited by email. We used the following criteria to select the participants: a) having completed or currently pursuing undergraduate or graduate studies in saxophone performance, b) having a minimum of 10 years of formal saxophone practice, c) having previous knowledge of the piece to be recorded. In the invitation, participants were inquired about the previous information. No statistical methods were used to predetermine the sample size, but the present sample is larger than previous analogous studies (e.g., 9 participants in Klein et al. 2022, 10 participants in Massie-Laberge et al. 2019).

Data collection	Motion data was collected using an optical 3D motion capture system and placing retroreflective markers in the body of the participants. Audio data was recorded with a Zoom H4N recorder. The researcher who conducted data collection was not blind to the experimental conditions and to the study's aims.<br>Recording sessions were individual and scheduled according to the participants' availability. An assisting technician was present in the sessions.
Timing	May 2021 - October 2021
Data exclusions	No data were excluded in the analyses - main results are based on the complete dataset.
Non-participation	Six participants declined participation a priori due to unavailability.
Randomization	Participants were not allocated into experimental groups. The musical excerpts and expressive conditions were randomized in the recording sessions.

Ecological, evolutionary & environmental sciences study design

All studies must disclose on these points even when the disclosure is negative.

Study description	*Briefly describe the study. For quantitative data include treatment factors and interactions, design structure (e.g. factorial, nested, hierarchical), nature and number of experimental units and replicates.*
Research sample	*Describe the research sample (e.g. a group of tagged Passer domesticus, all Stenocereus thurberi within Organ Pipe Cactus National Monument), and provide a rationale for the sample choice. When relevant, describe the organism taxa, source, sex, age range and any manipulations. State what population the sample is meant to represent when applicable. For studies involving existing datasets, describe the data and its source.*
Sampling strategy	*Note the sampling procedure. Describe the statistical methods that were used to predetermine sample size OR if no sample-size calculation was performed, describe how sample sizes were chosen and provide a rationale for why these sample sizes are sufficient.*
Data collection	*Describe the data collection procedure, including who recorded the data and how.*
Timing and spatial scale	*Indicate the start and stop dates of data collection, noting the frequency and periodicity of sampling and providing a rationale for these choices. If there is a gap between collection periods, state the dates for each sample cohort. Specify the spatial scale from which the data are taken*
Data exclusions	*If no data were excluded from the analyses, state so OR if data were excluded, describe the exclusions and the rationale behind them, indicating whether exclusion criteria were pre-established.*
Reproducibility	*Describe the measures taken to verify the reproducibility of experimental findings. For each experiment, note whether any attempts to repeat the experiment failed OR state that all attempts to repeat the experiment were successful.*
Randomization	*Describe how samples/organisms/participants were allocated into groups. If allocation was not random, describe how covariates were controlled. If this is not relevant to your study, explain why.*
Blinding	*Describe the extent of blinding used during data acquisition and analysis. If blinding was not possible, describe why OR explain why blinding was not relevant to your study.*

Did the study involve field work? ☐ Yes ☐ No

Field work, collection and transport

Field conditions	*Describe the study conditions for field work, providing relevant parameters (e.g. temperature, rainfall).*
Location	*State the location of the sampling or experiment, providing relevant parameters (e.g. latitude and longitude, elevation, water depth).*
Access & import/export	*Describe the efforts you have made to access habitats and to collect and import/export your samples in a responsible manner and in compliance with local, national and international laws, noting any permits that were obtained (give the name of the issuing authority, the date of issue, and any identifying information).*
Disturbance	*Describe any disturbance caused by the study and how it was minimized.*

Reporting for specific materials, systems and methods

We require information from authors about some types of materials, experimental systems and methods used in many studies. Here, indicate whether each material, system or method listed is relevant to your study. If you are not sure if a list item applies to your research, read the appropriate section before selecting a response.

Materials & experimental systems

n/a	Involved in the study
☒	☐ Antibodies
☒	☐ Eukaryotic cell lines
☒	☐ Palaeontology and archaeology
☒	☐ Animals and other organisms
☒	☐ Clinical data
☒	☐ Dual use research of concern
☒	☐ Plants

Methods

n/a	Involved in the study
☒	☐ ChIP-seq
☒	☐ Flow cytometry
☒	☐ MRI-based neuroimaging
